# Demand for health care service and associated factors among patients in the community of Tsegedie District, Northern Ethiopia

**DOI:** 10.1186/s12913-018-3490-2

**Published:** 2018-09-10

**Authors:** Tsegay Wellay, Measho Gebreslassie, Molla Mesele, Hailay Gebretinsae, Brhane Ayele, Alemtsehay Tewelde, Yodit Zewedie

**Affiliations:** 1 Department of Health system, School of Public Health, College of Health Sciences, Mekelle University, Mekelle, Ethiopia; 2 Department of human nutrition development, Institute of Public Health, College of Medicine and Health Sciences, University of Gondar, Gondar, Ethiopia; 3 Department of Public Health Emergency Management, Institute of Tigray Health Research, Mekelle, Ethiopia

**Keywords:** Demand of health care, Multinomial logit model, Tsegedie District, Modern health care service

## Abstract

**Background:**

Demand-side barriers are as important as supply factors in deterring patients from obtaining treatment. Developing countries including Ethiopia have been focusing on promoting health care utilization as an important policy to improve health outcomes and to meet international obligations to make health services broadly accessible. However, many policy and research initiatives focused on improving physical access rather than focusing on the pattern of health care service utilization related to demand side. Understanding of determinants of demand for health care services would enable to introduce and implement appropriate incentive schemes to encourage better utilization of health care services in the community of Tsegedie district, Northern Ethiopia.

**Methods:**

A community based cross sectional study design was conducted from March1–30/2016 in Northern Ethiopia. Systematic random sampling technique was used to select 423 participants from 2189 patients of the one-month census. A pretested and standardized semi-structured interviewer administered questionnaire was used to collect the data. The data were entered using Epi-info version 7 and analysed by STATA version 11. Multinomial logistic regression model was used to identify the determinants of demand for health care service.

**Results:**

A total of 423 (with a response rate of 98.3%) study participants were included in the study. The finding indicates that 72.5% (95%CI = 61.6, 81.1) of the participants demanded modern health care services. The multinomial logistic regression econometric model revealed that perceived severity of illness (β = 1.27; 95% CI = 0.74, 1.82), being educated household head (β = 0.079; 95% CI = 0.96, 1.74), quality of treatment (β = 0.99; 95% CI = 0.47, 1.5), distance to health facility β = 1.96; 95%CI = 0.11, 0.27), cost of treatment (β = − 1.99; 95% CI = 0.85, 3,13) were significantly and statistically associated with demand for health care service.

**Conclusion:**

This study revealed that in Tsegedie district, majorities (72.5%) of the patients demanded modern health care service. Distance to health care facility, user-fees, educational status of household, quality of service, and severity of illness were found to be significantly associated with demand for health care service.

Out of pocket, payments should be changed by prepayment schemes like community-based insurance than to depend on user fees and appropriate health information dissemination activities should strengthen to create awareness about modern care.

## Background

Demand for health care is characterized by the level of actual consumption of an individual incase of facing illness/injury, this consumption could differ in accordance with demand factors such as income, cost of care, education, social norms and traditions, and the quality and appropriateness of the services provided [1, 2]. It is multidimensional perspective that an individual making a decision in case of illness/injury as far as health care is concerned [3].

Developing countries have been focusing on promoting health care utilization as an important policy to improve health outcomes and to meet international obligations to make health services broadly accessible. However, many policy and research initiatives focused on improving physical access rather than focusing on the pattern of health care service utilization related to demand side [4].

In low-income countries, there is a clear direction to allocate scarce fiscal resources based on a clear understanding of how investments in the heath sector are going to affect demand. Likewise, to understand how changes in the pricing of public services, and investments in quality improvements, are going to affect consumer decisions about whether and where to seek health care [5, 6]. The occurrence of illness results household welfare loss through increased spending on health or reduced labor productivity and Ethiopia’s health sector is highly donor financed nearly to half(48.9%) and out of pocket (34%) of the national health expenditure [7, 8].

In developed countries due to the existence of insurance, many health care services has been provided at zero or low monetary prices, and the demand should be infinite or at least extremely high, However, in developing countries context under-utilization is generally more of a concern and lack of supply is considered as the main cause for under-utilization. But even when health facilities are available utilization rate has been low due different barriers from demand side which related to financial cost of treatment, travelling cost and quality of services [9, 10].

“The perception of health/illness relates to engaging in preventive behavior and self-care and the importance of self-perceived health status incorporates a clinical framework”, “analyzing the social assessment and treatment of disease, health conditions and the social environment of individuals and Study conducted in USA suggested self-perceived health is strongly correlated to the risk of dying” [11, 12]. studies based on behavioral approaches also called the willingness-to-pay (WTP) methods—have often tended to gross over the distributional implications of the demand estimates and the expenditures financing those public services [13].

As the policy priority area is to improving the health status of the population, there should be investigation in different factors that directly and indirectly affects the demand of the health care services. That is, necessary to analyze the demand for health care services by identifying the factors that affect individuals’ decisions to seek health care services and to choose among different providers [14].

Therefore, assessing the determinants of demand for health care services would enable to introduce and implement appropriate incentive schemes to encourage better utilization of health care services in Ethiopia. This will be helpful in identifying specific barriers in seeking health institution conditionally being sick/injury. In addition, to have information on sick individual preferences to different health care service providers in case of visiting modern health institutions.

Since health is an important component of human capital, “good health can substantially increase the capabilities of individuals to perform various activities, including income–generating ones; As a result, individuals demand good health” [15, 16].

Health, at the individual level, is mainly influenced by a variety of factors such as unobservable biological determinants, lifestyle choices, non–medical purchased inputs, purchased medical inputs (health care), and various socio–economic factors [17–19]. It is crucial to understand patients’ perceptions and expectations of the quality of care, because the perceived quality of health care services often influences the consumption behavior and patterns of health service [20]. “Study conducted in Kenya shows that quality of service offered at health facility significantly affects the demand for health care 6 times increase the likelihood of visiting modern health care service relative to self treatment” [21].

## Methods

The study was carried out in the community of Tsegedie woreda Tigray regional state, North Ethiopia from March 1–30/ /2016.; which is located 1512 km away from the capital city Addis Ababa. It has estimated total population of 126, 608, of these 63,937 are females and 18,928 are under five children and estimated number of households of the woreda based on the 2007 census conversion factor was about 28,774. Tsegedie has 1 general hospital, 1 primary hospital, 5 governmental health centers, 5 private medium clinics and 13 private drug stores.

### Theoretical and empirical framework

“The neoclassical economic theory of rational consumer and constrained utility maximization is the cornerstone of modern health care demand analysis. In this paradigm, people are assumed to drive utility directly from the health obtained from medical services. The model is based on the idea that ‘an individual choose the outcome that maximizes the utility gained from the choice” [22]. “This implies that given two alternatives A and B, and assuming there is no ties in utilities; a rational individual chooses alternative A if and only if U_A_ > U_B_ and vice versa. In the events of illness, the individual “*I”* is assumed to maximize utility (U) conditional on the consumption of health provided by provider “j”, subject to the budget constraint and health production function” [23, 24].

1 $$ \operatorname{Max}\ \mathrm{Uij}=\mathrm{U}\ \left(\mathrm{Hij},\mathrm{Cij}\right)+{\mathrm{e}}_{\mathrm{ij}}, $$

2 $$ \mathrm{Subject}\ \mathrm{to}:\mathrm{mi}=\mathrm{Pij}+\mathrm{Pc}\ \mathrm{Cij}\ \left(\mathrm{budget}\ \mathrm{constraint}\right) $$

3 $$ \mathrm{Hij}=\mathrm{Ho}+\mathrm{Qj}\ \left(\mathrm{X},\mathrm{Z}\right)\ \left(\mathrm{Health}\ \mathrm{Production}\right), $$

Where:

Hij -Is the post- treatment health status of the individual treated by provider *j.*

Cij -Is the consumption level composite goods after choosing provider *j.*

Eij- Is the random error term that captures the notion that efficacy of medical care is not perfect.

mi- Is the total income of the household.

Pij -Is the price of medical treatment from provider *j*. It includes both money and time costs.

Pc -Is the price of the composite consumption goods.

Ho -Is the initial health status of the individual.

Qj- Is the improvement in health status of the individual after the treatment by provider *j.*

The improvement in health status of an individual (Qj) is a function of a vector of individual characteristics (**Z**) provider characteristics (**X**) [25]. It follows that individual *i* will maximize the unconditional utility function (U*) which is given by

4 $$ {\mathrm{Uj}}^{\ast }=\max\ \left(\mathrm{Ui}1,\mathrm{Ui}2,---,\mathrm{Ui}\mathrm{j}+1\right) $$

Where.

Uij Is the utility function of care from provider j = 1, 2, − --, j + 1.

Solution to eq. (4) provides the health care alternative that yield the highest utility and to be chosen by an individual [25, 26]. Normalizing Pc to one and substituting eqs. (2) & (3) into eq. (1), we will get *conditional utility function* of provider *j* that can be written as

5 $$ \mathrm{Uij}=\mathrm{U}\ \left(\mathrm{Ho}+\mathrm{Qij}\ \left(\mathrm{X},\mathrm{Z}\right),\mathbf{m}\mathrm{i}-\mathrm{Pij}\right)+\mathrm{eij} $$

When the above utility function is quasi linear in Hij and Cij and these components are greater than zero, the indirect utility function is given by:

6 $$ \mathrm{V}=\mathrm{V}\ \left(\mathrm{P},\mathrm{H},\mathrm{Q}\ \left(\mathrm{X},\mathrm{Z}\right),\mathbf{m}\right)+\mathrm{e} $$

Equation (6) is the reduced form of the indirect utility function of alternative *j* and in most of the literature; it forms the basis of estimating health care demand functions. It conveys the message that the demand for health care depends on price, health status, the improvement in health status and individual and providers’ characteristics [27].

The indirect utility obtained by a patient from a given clinic, according to [28], can be derived from a multiplicative indirect utility specification and is given as:

7 $$ \ln\ \mathrm{V}={\mathrm{V}}^{\ast }+{\mathrm{e}}_{\mathrm{ij}} $$

Where V denotes the indirect utility gained by the individual i (*i* = 1, 2, − - -) from choosing j clinic type (j = 1, 2, − - -, J) and e is the random error term.

Furthermore, V can be decomposed into

8 $$ {\mathrm{V}}_{\mathrm{i}\mathrm{j}}={\mathrm{X}}_{\mathrm{i}\mathrm{j}}\beta +{\mathrm{z}}_{\mathrm{i}}{\upalpha}_{\mathrm{i}} $$

Where: X (=ln X 1, ln X 2, − - -, ln X k), the X k’s (k = 1, 2, − - -, k) are alternative (provider type) specific observable exogenous variables;

Β is a K × 1 vector of unknown parameters to be estimated;

Zi = (ln Zi1, ln Zi2, −--, ln ZiG), the ZiG’s (g = 1, 2, −---, G) are individual (patient) specific observable exogenous variables;

α_i_ = is a Gx1 vector for unknown parameters to be estimated [28].

Assuming IID (independently identically distributed) log-Weibull distribution for the disturbances e_ij_,the P_ij,_ the probability that individual *i* will choose clinic type j, according to [26], is

$$ {\mathrm{P}}_{\mathrm{ij}}=\frac{\exp\ \left({{\mathrm{V}}_{\mathrm{ij}}}^{\ast}\right)}{\sum_{j=1}^j\exp \left( Vij\ast \right)}(9) $$

Substituting eq. (8) in to Eq. (9), the probability that individual *i* will choose clinic type *j* is rewritten as

$$ \mathrm{Pij}=\frac{\exp\ \left({\mathrm{X}}_{\mathrm{ij}}\beta +{Z}_{\mathrm{j}\upalpha \mathrm{i}}\right)}{\sum \limits_{j=1}^j\exp \left(\mathrm{Xij}\beta \ast +\mathrm{Zi}\upalpha \mathrm{i}\right)}(10) $$

Assuming Yi be a random variable indicating the choice made, the probability of choosing provider j, according to [27] and [29], is:

$$ \mathrm{pij}=\mathrm{prob}\left(\mathrm{Yi}=\mathrm{j}\right)=\frac{\exp\ \left({\mathrm{X}}_{\mathrm{i}\mathrm{j}}\beta +{\mathrm{Z}}_{\mathrm{j}\upalpha \mathrm{i}}\right)}{1+{\sum}_j^j\exp \left({\mathrm{X}}_{\mathrm{i}\mathrm{j}}\beta \ast +{\mathrm{Z}}_{\mathrm{i}\upalpha \mathrm{i}}\right)}(11) $$

Furthermore the likelihood for this problem is lnLi = Dij ln**p**r(Yi = j), where Dij is a dichotomous variable that takes on the value 1 if individual *i* chooses alternative *j* (i.e., if Yi = j) and 0 if he/she do not choose it [26, 27] and [29].

### Estimation & Variable

The analysis for demand of health care provider choice in Tsegedie district, northern Ethiopia was carried out using the multinomial logit model. Estimation of the multinomial logit model specified in equation [29] above is undertaken using a statistical package called STATA. Model fitting was checked by maximum likelihood model, Multicollinearity was checked by Durbin-Watson that is the variable inflation factor.

The dependent variable is choice of specific health care providers. The alternative health care providers are Public health care providers, Private health care providers and self-treatment or not consulted modern care. Our response variable, health care provider choice is going to be treated as categorical under the assumption that the choice of health care provider has *no* natural ordering.

$$ \mathrm{Health}\ \mathrm{care}\ \mathrm{provider}\ \mathrm{choice}=\left\{\begin{array}{l}1\ \mathrm{if}\ \mathrm{consulted}\ \mathrm{public}\ \mathrm{health}\ \mathrm{care}\ \mathrm{provider}\\ {}2\ \mathrm{if}\ \mathrm{consulted}\ \mathrm{private}\ \mathrm{health}\ \mathrm{care}\ \mathrm{provider}\\ {}3\ \mathrm{if}\ \mathrm{no}\ \mathrm{consulted}\ \mathrm{modern}\ \mathrm{health}\ \mathrm{care}\ \mathrm{provider}\ \left(\mathrm{self}\hbox{-} \mathrm{treatment}\right)\end{array}\right. $$

The independent variables include different attributes of the individual patient, socioeconomic and the health care providers.

### List and description of explanatory variables

Agehhd = A continuous variable for the age of household head.

Agept = A categorical variable (1 = < 5 years old, 2= > =5) years old.

Male = A nominal variable 1for male and 2 for female.

EducaHHd2 = educational level of household head (1 = not educated, 2 primary, and secondary and above.

HHsize = nominal variable (1 = < 5 and 2= > = 5).

Marital2 = nominal categorical variable (1 = married, 2 not married, 3 divorced and 4 for widowed).

HHincome2 = household monthly income (1 = 0-1000birr, 2 = 1001–2500 birr, 3 = 2501–4500 birr and 4 = 4500 birr).

Educapt2 = educational; level of patient (1 = not educated, 2 = primary school, and 3 = secondary school and above).

Severity2 = nominal variable (1 = mild illness and 2 = severe illness).

Sexpt2 = nominal variable (1 = male, 2 = female).

Costoftreat2 = categorical variable for cost of treatment in birr (1 = < 50, 2 = 50–100, 3 = 201–300 and 4= > 300.

Qualiofser2 = perceived quality of treatment categorical (1 = poor and 2 = good).

DistantoHF = continuous variable the nearest health facility, in kilometers.

The study utilized community-based cross-sectional study design with quantitative data collection method. The study population included all households experienced illness within 4 weeks prior to the study period in the community of Tsegedie woreda, Northern, Ethiopia. Patients admitted for treatment during the data collection period were excluded from the study, may not give reliable information as far as they did not finish their treatment for measuring quality of treatment outcome.

The sample size was calculated using single population proportion formula with the following assumptions: proportion 50%, which was appropriate to get maximum sample size than the sample size calculated using Epi-info software considering 80% power. Using 5% margin of error at 95% confidence level, the sample size was 423 after considering 10% non-response rate. All kebeles of the woreda were included in the study after 1 month of census was done and 2189 patients were found. Therefore, 423 patients were selected using systematic random sampling technique after proportional allocation for each kebelle has been done. The independent variables were included from similar studies conducted elsewhere [24, 30–32].

The questionnaires were prepared by reviewing relevant literatures and adapting from previously done articles [30]. Pre- test was done on 10% of the subjects outside the study area at Welkaite district. Data were collected by pre-tested, pre-coded and interviewer-administered questionnaires. The collected data were cleaned, coded, entered into Epi-info version 7 software, transferred, and analyzed using STATA computer soft ware package version 11. Summary statistics of socio demographic variables were presented using frequency tables and graphs. The data analyzed using multinomial logistic regression to determine the effect of various factors on the outcome variable. Variables were entered into multiple logistic and multinomial logit regression models to control confounding effect.

The results were presented in the form of tables, figures and text using frequencies and summary statistics such as standard deviation, mean, and percentage to describe the study population in relation to relevant variables. The degree of association between dependent and independent variables were assessed using log odds ratio with 95% confidence interval and *p*-value 0.05.

The study was reviewed and approved by Institution Research Review Board of, Institute of Public Health, College of Medicine and Health science, and University of Gondar. Official letter of permission was also obtained from Tigray Regional Health Bureau and Tsegedie Health Office. The purpose and the importance of the study were orally explained and written consent was obtained from each participant. Moreover, confidentiality of the information was assured by using anonymous questionnaires and by keeping the data in a secured place.

## Results

A total 423 households were participated in the study with a response rate of 98%. Majority of household heads 289(69.14%) were males and 327(78.2%) were Tigrian in ethnicity. More than 90% of households were orthodox Christian.

About 285 (68.2%) of respondents had four or more family members and the mean of family size was four members (SD± 2.7). More than half 219 (52%) of the respondents were illiterates, and 177 (42.3%) were farmers in occupation and 91 (21.7%) were daily laborers. Concerning marital status majority 285(68.2%) of the household heads were married (Table 1).

**Table 1:** Socio-demographic characteristics of households having ill/injured member of the family in the community of Tsegedie woreda, northern Ethiopia, April, 2016 (*n* = 418)

Variables	Description	Number (%)
Sex of household head	Male	289(69.1)
	Female	129(31.9)
Ethnicity:	Tigray	327(78.2)
	Amhara	91(22.8)
Religion:	Orthodox	387(92.6)
	Muslim	29 (6.9)
	Others	2(0.5)
Marital status:	Married	286(68.4)
	Not married	32 (7.7)
	Divorced	80(19.1)
	Widowed	20 (4.8)
Occupational status of HHd:	Farmer	177(42.3)
	Merchant	57(13.6)
	Civil servant	45(10.8)
	Daily laborer	108(25.8)
	Private employed	27 (6.5)
	Housewife	4 (0.9)
Educational status of HHd:	No formal education	219 (52.4)
	Primary school	128 (30.6)
	Secondary school	38 (9)
	Above secondary school	33 (7.9)

The frequency table below sows that more than half 106 (51.2%) of patients, whose household heads with no education consult medical care in the public health care provider. Nevertheless, 76 (66%) of patients whose household heads were with no education did not Consult medical treatment (Table 2).

**Table 2:** Health care provider chosen by education level of household head and Patient, at Tsegedie woreda, northern Ethiopia, April 2016

	Health care provider chosen		
	Public	Private	No-care
	Number (%)	Number (%)	Number (%)
Household head educational status			
No formal education	106 (51.2)	38 (39.6)	75 (65)
Primary	66 (31.8)	36(37.8)	26 (22.6)
Secondary	15 (7.2)	13 (13.5)	10 (8.7)
Above secondary	20(9.7)	9(9.4)	4(3.5)
Educational status of patient			
No formal education	106 (51.2)	41 (42.7)	76 (66)
Primary	65 (31.4)	36 (37.5)	24 (20.9)
Secondary	21 (10.1)	13 (13.5)	13 (11.30)
Above secondary	15(7.2)	6(6.50)	2(1.70)

Households were divided in to four quartile representing income group ranging from quartile one (lowest income) to quartile four (highest income) based on stated monthly income. The result survey revealed that the higher household income associated with higher probability of seeking medical treatment in times of illness. Concerning to the choice of health care provider, households preferences seems to shift from public health facilities to those of private ones as there income level rises, especially when we compare with the lowest income group(*p* = 0.033) (Table 3).

**Table 3:** Medical care seeking behavior and provider related choice by income group (asset), at Tsegedie woreda, northern, Ethiopia April 2016

Income quartiles in birr		Option chosen			Total
		Public	Private	Not treated	
0–1000	Count	86	34	58	178
	% within income group	48.2	19.1	32.8	100
1001–2500	Count	78	29	39	146
	% within income group	54.2	20	26.7	100
2501–4500	Count	26	19	9	54
	%within income group	48.1	35	16.6	100
> 4500	Count	15	16	9	40
	%within income group	39.4	40	23.6	
Total		205	98	115	418

Cross tabulation of the responses indicates that, given the type of illness that made patients visit physician, an increase in cost of treatment cause a decline in the number of patients who consulted public health care provider, whereas the for private health care provider first increase then decline. The result suggests that an increase in cost of treatment may improve the probability of consulting private health care provider relative to public provider. This indicates that presence of correlation between better quality and higher cost of treatment in private health care provider (*p* < 0.0001).

Concerning to the perceived quality of health care, about 86.5% and 13.5% of the respondents judged the quality of public health care providers as good and poor respectively. Similarly, the perceived quality of the private health care providers was good (94.7%) and poor (5.3%).

The study revealed that 303(72.5%) (95%CI; 63.8, 81.6) of the study participants consulted modern health care service. Among those who visited modern health care facilities, 49.5 had visited public health care providers. However, abound 27.5% of the respondents did not visit modern health care services. Majority of the patients who consulted public owned health care providers explained the availability of drugs, lower cost of treatment and nearness of the providers as their main reasons for consulting them. However, short waiting time and quality of treatment were the main reasons for choosing private health care providers.

### Factors associated with demand of health care service

The dependent variable used was health care choice represented by “Type”, while the independent variables are individual, household, and access. The coefficients of each variable reflect the effect of a change in each of the variables on the probability that the individual will consult medical care and choose a certain provider relative to no-care (self-treatment).

The multinomial logit of being educated household head relative to less educated household head is 0.079 (95% CI 0.966, 1.124) and 0.966 (95%CI 0.050, 3.438) units higher to visit public health care provider and private health care provider to self treatment respectively, given all other predictor variables in the model are held constant.

Regarding to severity of illness, as the severity of illness increases, the log odds of consulting public and private health care providers increased by 1.27(95%CI 0.74,1.80) and 1.82(95%CI 1.45, 2.502) respectively in favor of self treatment.

As the perceived quality of treatment increases, the log odds of opting to the public and private health care providers increased by 0.989(95%CI 0.472, 1.507) and 1.154(95%CI 0.624, 1.683) times relative to self-treatment respectively. Similarly, as the cost of treatment of private health care provider increases; the multinomial logit of consulting private health care providers expected to decrease by 2.510 times relative to the self-care treatment.

Age of patient is statistically significant and negatively associated with health care demand. If the age of the patient were to increase by one year, the multinomial log-odds of consulting public health care provider relative to self- treatment would be expected to decrease by 0.63(95%CI-1.24, −.008) times. Similarly, as distance to health facility increases by 1 km, the probability to opt public and private health care providers would be expected to decrease by 0.13 and 1.96 times respectively relative to self-treatment (Table 4).

**Table 4:** The log-odds for Private and public health care providers relative to not treated Multinomial Logistic Regression Results of patients at Tsegedie woreda, northern Ethiopia, 2016

Health care Providers	Coef.	Std. Err.	Z	*p* > |z|	[95% Conf. Interval]	
Public Health Care Providers						
Agehhd	−0.005	0.016	−0.34	0.737	−0.037	0.026
Male1	0.774	0.490	1.58	0.115	−0.187	1.735
EducaHHd2	0.079*	0.533	0.15	0.043	0.966	1.124
EducaHHd3	−0.153	0.757	− 0.20	0.839	−1.638	1.330
HHsize	0.160	0.106	1.50	0.133	−0.048	0.369
Marital2	−0.915	0.670	−1.36	0.172	−2.229	0.399
HHincome2	0.083	0.303	0.27	0.784	−0.511	0.677
HHincome3	0.034	0.510	0.07	0.946	−0.966	1.035
HHincome4	−0.512	0.527	−0.97	0.331	−1.545	0.520
Educapt2	1.010	0.542	1.86	0.062	−0.051	2.073
Educapt3	0.627	0.680	0.92	0.356	−0.706	1.961
Educapt4	1.567	1.378	1.14	0.256	−1.134	4.268
Severity2	1.272**	0.271	4.70	0.000	0.741	1.803
Agept2	−0.625*	0.314	−1.99	0.047	−1.242	−0.008
Sexpt2	0.332	0.277	1.20	0.232	−0.212	0.876
DistantoHF	0.132**	0.036	3.60	0.000	0.060	0.204
Qualiofser2	0.989**	0.263	3.75	0.000	0.472	1.507
Costoftreat2	1.023	6.031	0.02	0.987	−1.562	1.608
costoftreat3	1.640	1.484	0.01	0.988	−2.951	2.232
costoftrea4	−0.708	1.179	−0.60	0.548	−3.019	1.601
costoftrea5	1.086	1.381	0.01	0.994	−2.512	3.684
_cons	−1.660	0.760	−2.18	0.029	−3.150	−0.170
Private Health Care Providers						
AgeofHHd	0.017	0.019	0.89	0.372	−0.020	0.055
SexofHHd1	0.620	0.568	1.09	0.275	−0.493	1.735
EducaHHd2	0.630	0.603	1.05	0.296	−0.551	1.813
EducaHHd3	1.744*	0.864	2.02	0.044	0.050	3.438
EducaHHd4	0.868	1.318	0.66	0.510	−1.716	3.453
HHsize	0.242	0.125	1.94	0.053	−0.002	0.488
Marital2	−0.326	0.778	−0.42	0.675	−1.851	1.199
Marital3	−0.794	0.637	−1.25	0.213	−2.043	0.455
Marital4	−0.264	0.852	−0.31	0.756	−1.936	1.406
HHincome2	−0.137	0.388	−0.35	0.724	−0.897	0.623
HHincome3	0.734	0.569	1.29	0.197	−0.382	1.852
HHincome4	0.506	0.573	0.88	0.377	−0.617	1.630
Educapt2	0.779	0.605	1.29	0.198	−0.407	1.967
Educapt3	−0.134	0.808	−0.17	0.868	−1.718	1.449
Educapt4	0.416	1.546	0.27	0.788	−2.615	3.447
Severity2	1.824**	0.346	5.27	0.000	1.145	2.502
Ageofpt2	0.264	0.375	0.70	0.481	−0.471	1.001
Sexofpt2	−0.194	0.335	−0.58	0.562	−0.852	0.462
DistantoHF	−0.196**	0.041	4.68	0.000	−0.278	−0.114
Qualiofser2	0.989**	0.263	3.75	0.000	0.472	1.507
costoftrea2	1.699	6.031	0.02	0.987	−1.886	1.285
costoftrea3	1.621	1.485	0.02	0.987	−2.970	1.213
Costoftrea4	−2.510*	1.118	2.24	0.025	−4.702	−0.317
costoftrea5	1.948	1.381	0.01	0.992	−3.649	3.546
_cons	−5.072	0.982	−5.17	0.000	−6.997	−3.148

Not treated (base outcome) **significant at 1% and less, *significant at 5% and less. HHd = household head

The variables were statistically predicted at; LR chi2 (44) = 122.01 Log likelihood = − 372.8138, Pseudo R2 = 0.1406

## Discussion

The finding of this study showed that 72.5% patients consulted modern health care. This finding is almost similar with the studies conducted in Mekelle town (79%) [33] and Kenya (70%) [30]. This result shows that the demand of patients for modern health services in Northern Ethiopia is low. This implies, still, significant number of patients either is not getting health services or is consulting traditional healers. The observed low demand for health care might be due to community-based health insurance or other prepayment schemes are not yet implemented in the community.

The multinomial logit regression analysis revealed that, having higher educational level (secondary school and above) of household heads increases the probability of seeking modern medical care than with no education. This finding was consistent with studies conducted in Tanzania [5], Kenya [34] and urban Ethiopia [35]. This might be because the educated people may have better awareness about the importance of modern medicine. Similarly, perceived severity of illness was found to be another factor statistically associated with demand for health care service. This study revealed that as the duration of illness increases, the probability of consulting modern health care service increased related to mild illness. This result is supported with the studies conducted in urban Ethiopia [35] and rural Ethiopia [36]. Patients’ perception regarding the seriousness of illness plays a significant role in their decision of consulting in both public and private modern medical treatment related to mildness of illness.

The perceived quality of health care services was statistically and significantly associated with the patients’ demand for health care services. As the quality of treatment increases, the probability of opting to public and private health care providers increases relative to self-treatment. This finding is similar with the studies conducted in Uganda [37], Kenya [34] and rural Tanzania [5]. This might be due to the reason that high-perceived quality of care in the public, private health care facilities have the potential to attract patients, and it is correlated with high patient satisfaction.

Increasing user charges decreases the likelihood of seeking health care from the modern health care provider relative to self-treatment. The result of this study is consistent with studies conducted in urban Ethiopia [35],Kenya [38] and cote D’Ivore [39]. This might be because demand for health care is negatively affected by lower income and high price of health care. However, this result is not supported by a study conducted in Philippines [40]. The observed difference might be due to the difference in the implementation of financial protection mechanisms and socio-economic level between the two countries.

Distance to health facilities has statistical and significant association with demand for health care services. The multinomial logit model showed that as distance to health facility increases, the probability to opt self-treatment increases. This finding concurs with the study conducted In Kenya [41], Coted’ivore [39] and Benin [4]. This may be paying some cost to travel to the source of treatment as opposed to seeking self-treatment.

This study has limitations particularly due to recall bias because the patients were expected to tell us a one-month history of illness and related costs. However, we have made tried to associate with special events to reduce the possible recall bias.

## Conclusion

This study showed that more than one-fourth patients did not demand modern care. Educational status of household head, distance to health facility, cost of treatment, severity of illness, and quality service were independent predictors of demand for health care service. The Regional Health Bureau, Woreda Health Office and other relevant stakeholders need to design and implement prepayment schemes such as community based health insurance to increase the patients demand for modern health care services. Similarly, the Woreda Health Office needs to work to improve the awareness of patients on modern health care services.

## Abbreviations

CI: Confidence Interval
EDHS: Ethiopian Demographic and Health Survey
FIML: Full Information Maximum Likelihood
FMOH: Federal Ministry of Health
HSDP: Health Sector Development Programme
IIA: Independent Irrelevant Alternatives
MLM: Multinomial Logit Model
NGOs: Non-Government Organizations
NHA: National Health Account
OR: Odds Ratio
WHO: World Health Organization
